# Diagnostic and Therapeutic Challenges of Cardiac Metastasis in Advanced Malignancies: A Case Series and Literature Review

**DOI:** 10.1155/crom/7374561

**Published:** 2025-05-24

**Authors:** Moath Albliwi, Aravinthan Vignarajah, Nishanthi Vigneswaramoorthy, Ayham Mohammad Hussein, Asfand Yar Cheema, Shimoli Barot, Gautam Shah

**Affiliations:** ^1^Department of Internal Medicine, Cleveland Clinic Foundation, Cleveland, Ohio, USA; ^2^Department of Internal Medicine, SUNY Upstate Medical University Hospital, Syracuse, New York, USA; ^3^Faculty of Medicine, Al-Balqa'a Applied University, Salt, Jordan; ^4^Department of Hematology and Oncology, Cleveland Clinic Foundation, Cleveland, Ohio, USA; ^5^Department of Cardiology, Cleveland Clinic Foundation, Cleveland, Ohio, USA

## Abstract

**Background:** Cardiac metastases, though more common than primary cardiac tumors, remain under-recognized due to their often subtle clinical presentation. These tumors can lead to life-threatening complications, and their diagnosis is typically delayed.

**Objective:** This paper is aimed at reviewing two distinct cases of metastatic cardiac tumors, shedding light on diagnostic challenges, clinical presentations, and management approaches.

**Methods:** We present two cases of patients with metastatic melanoma and undifferentiated malignant spindle cell neoplasm, respectively. Diagnostic imaging, including echocardiography and PET scans, was used to identify the cardiac masses, and biopsy results provided histopathological confirmation. Treatment plans involved systemic immunotherapy, chemotherapy, and surgical resection.

**Results:** In both cases, cardiac metastases were detected through advanced imaging, despite the patients presenting with nonspecific symptoms like abdominal pain and shortness of breath. The metastatic tumor in one patient responded to immunotherapy before surgical excision, while the other patient, in advanced stages, opted for supportive care.

**Conclusion:** Cardiac metastasis should be considered in cancer patients who present with unexplained cardiac symptoms. A multidisciplinary approach, including imaging and biopsy, is crucial for accurate diagnosis. Despite aggressive treatment, the prognosis remains poor, emphasizing the need for early detection and better therapeutic strategies.

## 1. Introduction

Metastatic disease in the heart is more prevalent than primary cardiac malignancies [1–4]. The clinical presentation of cardiac metastases is often nonspecific and highly variable, depending on the anatomical location and extent of metastatic burden within the heart [2, 3]. Although most cardiac metastases are clinically silent and only diagnosed postmortem, those that manifest clinically can be difficult to distinguish from other cardiovascular conditions. The diagnosis of cardiac metastasis may be confused with more common causes of cardiac symptoms, like benign cardiac masses and thrombi. Therefore, a comprehensive diagnostic evaluation is essential, including echocardiography (ECHO) and advanced cardiac imaging techniques [4, 5]. Despite these methods, a definitive diagnosis often requires a tissue biopsy, which can be challenging to perform. Furthermore, treatment guidelines remain vague and nonspecific. Metastatic cardiac tumors typically carry a poor prognosis due to systemic tumor involvement. Here, we report two cases with cardiac metastasis secondary to melanoma and sarcoma and provide a review of the literature on metastatic cardiac malignancies.

## 2. Case Presentation

### 2.1. Case 1

A 43-year-old female presented to the emergency room (ER) with low-grade fever (37.8°C) and severe localized stabbing right upper quadrant abdominal pain, rated 8/10 in intensity. Her past medical history was significant for a remote spitz nevus, which was excised. In the ER, she was tachycardic (heart rate 100 bpm) with normal blood pressure (124/74 mmHg) and oxygen saturation (99% on room air). No skin lesions were identified on physical exam. Laboratory results revealed normocytic normochromic anemia with a hemoglobin of 10.6 g/dL, elevated alkaline phosphatase (285 U/L), aspartate aminotransferase (AST) (45 U/L), and alanine aminotransferase (ALT) (43 U/L) with low albumin (3.7 g/dL).

Abdominal ultrasound, performed in August 2023, revealed two complex cystic lesions in the right hepatic lobe measuring 9.4 × 12.2 × 10.2 and 6.6 × 6.0 × 6.8 cm. Histopathological analysis confirmed the masses to be metastatic melanoma. A positron emission tomography (PET) scan in September 2023 was done, which showed no focal FDG-avid lesions in the head and neck. There was a small right pleural effusion but no concerning lymph nodes in the chest. The scan confirmed a large, intensely hypermetabolic lesion in the right liver, about 11 cm, consistent with known metastatic melanoma. No abnormal lymph nodes were seen in the abdomen or pelvis. However, there were multiple areas of active metastatic disease in soft tissue and bone, most notably in the left lower extremity. A superficial lesion was also noted on the left mons pubis, and dermatology follow-up was recommended. No heart involvement was seen on the PET scan.

**Figure 1:**
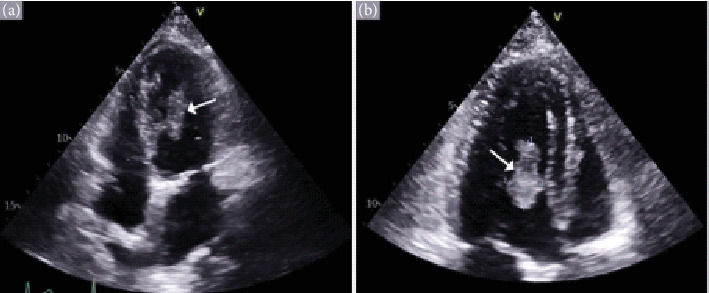
(a, b) Mobile left ventricular mass measuring 1.09 × 0.94 cm, originating from the mid anterolateral segment, close to the body of the papillary muscle.

The patient subsequently began systemic immunotherapy, receiving Cycle 1 of ipilimumab at 3 mg/kg and nivolumab at 1 mg/kg. One month after starting the immunotherapy, she returned to the ER with abdominal pain, fever, and palpitations. She was hemodynamically stable except for a heart rate of 115 bpm. Laboratory findings showed persistently elevated liver function tests, but blood cultures were negative. An electrocardiogram (EKG) revealed sinus tachycardia. She was treated with analgesics and antipyretics, although her tachycardia and low-grade fever persisted, which prompted further investigation with an ECHO. It demonstrated a mobile left ventricular mass measuring 1.09 × 0.94 cm, originating from the mid anterolateral segment, close to the body of the papillary muscle (Figure 1). It was confirmed through echocardiographic findings and biopsy, which identified the mass as metastatic melanoma. A multidisciplinary oncology consultation recommended continuation of the systemic immunotherapy regimen after further evaluation.

Approximately 2 months after initiating immunotherapy, the patient developed immune-related hepatitis. The patient received steroids and mycophenolate mofetil, and her immunotherapy regimen was de-escalated to nivolumab monotherapy. Serial ECHOs demonstrated progressive enlargement of the left ventricular mass despite ongoing immunotherapy. Because of this, the patient had a surgery “surgical excision of a left ventricular tumor” in March 2024. A follow-up ECHO confirmed the absence of the mass postoperatively.

### 2.2. Case 2

An 83-year-old lady presented to the ER for worsening shortness of breath and bilateral lower extremity edema. Her medical history was significant for hypertension, hyperlipidemia, coronary artery disease with prior stenting of the left anterior descending coronary artery, heart failure with preserved ejection fraction, chronic kidney disease, and anemia, for which she was receiving erythropoietin therapy. On presentation, she had a blood pressure of 154/56 mm Hg, a heart rate of 65 bpm, a temperature of 98.1°F, and a respiratory rate of 16 bpm, saturating 92% on room air. Clinically, the patient presented with shortness of breath, elevated jugular venous pressure (JVP), and bilateral lower limb edema, supporting a diagnosis of heart failure exacerbation. Laboratory results revealed normocytic normochromic anemia with hemoglobin levels of 9.4 g/dL, as well as elevated levels of d-dimer (1340 ng/mL), brain natriuretic peptide (25,000 pg/mL), high-sensitivity troponin (26 ng/L), and alkaline phosphatase (231 U/L). EKG and telemetry revealed a normal sinus rhythm with frequent runs of nonsustained ventricular tachycardia. She was diagnosed with acute heart failure exacerbation and was admitted to the cardiac step-down unit for further management.

**Figure 2:**
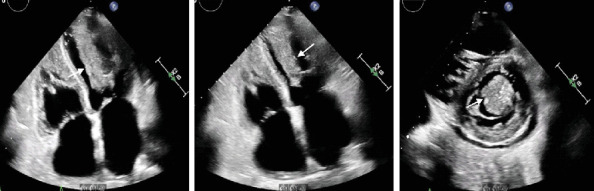
Fixed mass in the left ventricular cavity measuring 4.3 × 2.5 cm with severe mitral regurgitation secondary to restriction of the papillary muscle movement by the tumor.

An ECHO performed during hospitalization identified a large, fixed mass in the left ventricular cavity measuring 4.3 × 2.5 cm with severe mitral regurgitation secondary to restriction of the papillary muscle movement by the tumor (Figure 2). A computed tomography (CT) scan of the chest, performed to rule out pulmonary embolism, found multiple solid pulmonary nodules, mediastinal lymphadenopathy, and a 4.5 × 2.5 cm adrenal mass. CT scan of the abdomen and pelvis revealed a mass-like mucosal thickening in the small bowel (4.2 × 2.3 cm), mesenteric lymphadenopathy, hepatic masses, bilateral adrenal masses, a left kidney lesion, and an enlarged right retrocecal lymph node. An imaging-guided biopsy of the left adrenal mass confirmed an undifferentiated malignant spindle cell neoplasm.

**Figure 3:**
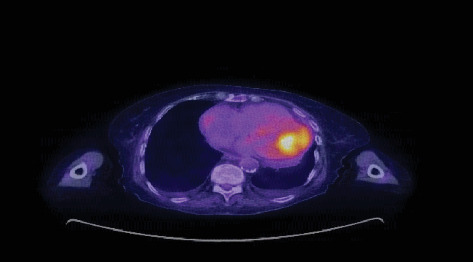
Hypermetabolic mass in the left ventricle.

Subsequent PET scan revealed hypermetabolic nodules in the lungs bilaterally, a hypermetabolic mass in the left ventricle (Figure 3), and hypermetabolic lesions in the liver, adrenal glands, celiac, and para-aortic nodules in the abdomen and pelvis. Brain imaging with both CT and magnetic resonance imaging (MRI) demonstrated two enhancing lesions in the left frontal lobe with vasogenic edema, consistent with metastatic disease. Given the extensive metastatic disease burden and in alignment with her preferences and goals, the patient decided to forgo any further surgical or systemic therapies and transitioned to hospice care.

## 3. Discussion

This case series represents two distinct cases of cardiac metastases in the setting of advanced malignancy. While the primary tumor origin and presentation differ, both cases illustrate the diagnostic and therapeutic challenges posed by metastatic cardiac tumors, an uncommon yet serious complication of cancer.

### 3.1. Prevalence

Cardiac metastases are significantly more prevalent than primary cardiac tumors [1, 4]. While primary cardiac tumors are rare, cardiac metastases are increasingly detected due to advancements in imaging techniques and the prolonged survival of cancer patients [1]. Autopsy studies have revealed that the incidence of cardiac metastases ranges from 2.3% to 18.3% [2]. Notably, the prevalence of cardiac metastases differs significantly between individuals without known malignancies and those with existing cancers. In the general population without known malignancies, the prevalence of cardiac metastases observed during autopsies ranges from 0.7% to 3.5% [5]. However, this figure increases to 9.1% in individuals with known malignancies [5].

Although any malignancy has the potential to metastasize to the heart, certain cancers exhibit a higher propensity. Notably, melanoma demonstrates a particularly high propensity for cardiac involvement, with 28%–65% of metastatic melanoma patients experiencing cardiac involvement [4, 6]. Other common primary cancers for the origin of cardiac metastasis include lung cancer (36%–39%), hematologic malignancies (10%–21%), breast cancer (10%–12%), and ovarian cancer (10%) [4, 5]. Other cancers with the potential to metastasize to the heart include gastroesophageal, skin, kidneys, thymus, and pancreatic cancers [3, 7, 8]. Conversely, sarcomas rarely metastasize to the heart, comprising approximately 5.5% of secondary cardiac tumors [9, 10] (Table 1).

Cancer can metastasize to the heart through four main pathways: direct extension, lymphatic spread, hematogenous spread, and transvenous extension. Oftentimes, the route of metastasis determines the specific cardiac location of the tumor. Lymphatic spread, the most common route, involves the migration of cancer cells through lymph vessels and nodes to reach the heart, primarily affecting the pericardium and epicardium [11]. Hematogenous spread occurs when cancer cells invade the bloodstream and travel to the heart. This typically results in myocardial or endocardial involvement, as seen in both cases [12]. This pathway is common for melanoma, lymphoma, and sarcoma [5].

Direct extension occurs when a nearby tumor, such as lung or breast cancer, invades the heart directly, often affecting the pericardium, the most common site of cardiac metastasis, involved in 64%–69% of cardiac metastases [2, 5]. Tumor spread to the pericardium often initially causes pericarditis, followed by the development of malignant pericardial effusions, which can be serosanguineous or hemorrhagic [2].

Approximately two-thirds of cardiac metastases affect the pericardium, while about 34.2% and 31.8% involve the epicardium and myocardium, respectively. Endocardial involvement is less common, occurring in only 5% of cases [2]. Finally, transvenous extension involves a tumor growing into a major vein, like the inferior vena cava, and extending directly into the heart, often the right atrium [13]. Renal cell carcinoma and hepatocellular carcinoma are notably associated with this form of metastasis [14].

### 3.2. Clinical Presentation

Cardiac metastasis often remains asymptomatic and is diagnosed most frequently during autopsy [5, 15]. Occasionally, when symptoms do occur, it generally is a sign of advanced disease [5]. When symptoms arise, they depend on the tumor burden and the location of cardiac metastasis, making diagnosis challenging. Patients may experience systemic symptoms like fatigue, fever, chills, night sweats, weight loss, joint pain, loss of appetite, and petechiae [16]. Cardiac-specific symptoms from the metastases depend on the cardiac structure involved rather than the source of the primary tumor. Since the pericardium is the most common site of cardiac metastases, symptoms from pericardial syndromes remain the more common way of presentation. Patients may present with pericarditis, signs and symptoms from pericardial effusions, or signs of constrictive pericarditis [17, 18]. Epicardial, myocardial, and endocardial metastasis can result in symptoms based on their location, like conduction abnormalities (heart blocks and atrial fibrillation), valvular dysfunction, myocardial dysfunction, and impede the flow of blood through cardiac chambers to intracavitary tumor growth. This can result in a decrease in cardiac output, symptoms of poor perfusion, and even cardiogenic shock. Rarely, presentation can even mimic acute coronary syndromes without coronary artery involvement [17].

### 3.3. Diagnostic Testing

Diagnosing cardiac metastasis typically requires a combination of diagnostic modalities. EKG findings are often nonspecific, with potential abnormalities such as low voltage, ischemia, heart blocks, and arrhythmias [19]. However, localized and prolonged ST elevation in the absence of ischemic symptoms can be highly specific for cardiac metastasis, especially in individuals with a known malignancy [5]. ECHO is frequently the initial imaging test, providing valuable information regarding tumor size, location, mobility, and pericardial invasion [20]. It also helps differentiate metastasis from other cardiac conditions like pericardial effusion or thrombus [15]. Cardiac magnetic resonance (CMR) imaging offers detailed information on tumor morphology and extent, including the involvement of surrounding structures [21], and aids in surgical planning. CT scans of the chest can identify cardiac and mediastinal tumors [22], while PET scans detect metabolically active tumors [23]. In addition to imaging, blood tests and chest x-rays may reveal abnormalities suggestive of metastasis [24]. Ultimately, a definitive diagnosis often relies on biopsy, which allows for histological confirmation of metastasis and identification of the primary cancer [25].

### 3.4. Treatment

Treatment for metastatic cardiac involvement focuses on symptom management, quality of life improvement, and potential disease control. The optimal approach depends on factors such as primary cancer type, extent of cardiac involvement, and the patient's overall health status. Systemic therapies, including chemotherapy, aim to eliminate or inhibit cancer cells, which can potentially reduce tumor size and alleviate symptoms. Radiation therapy employs high-energy rays to selectively damage cancer cells, particularly when surgical interventions are not feasible. Targeted therapies, focusing on specific molecules that drive cancer growth, can inhibit disease progression in certain cancer types. Additionally, immunotherapy boosts the immune system's ability to recognize and attack cancer cells. In certain cases, ablative treatments like stereotactic body radiation therapy (SBRT) or radiofrequency ablation (RFA) may be considered. Surgical resection, while complex due to tumor location, can be an option for symptom relief or complication management [26]. This is particularly relevant for cardiac angiosarcoma, which necessitates specialized surgical expertise [18].

#### 3.4.1. Case 1

The use of systemic immunotherapy, including ipilimumab and nivolumab, initially appeared promising but was complicated by an immune-related adverse event of hepatitis. Immunotherapy, particularly immune checkpoint inhibitors, has revolutionized the treatment of metastatic melanoma, improving survival outcomes. However, immune-related toxicities, like the hepatitis experienced by this patient, pose a significant challenge in its management. Despite immunotherapy, the patient's left ventricular mass continued to increase in size, necessitating surgical excision. This emphasizes the need for a multidisciplinary approach, where systemic therapy is combined with surgical intervention in certain cases, particularly when symptomatic cardiac metastasis is present.

#### 3.4.2. Case 2

Undifferentiated malignant spindle cell neoplasms are rare and often have a poor prognosis, particularly when associated with widespread metastatic disease, as seen in this patient. The large left ventricular mass further exacerbated the patient's heart failure, leading to a decision to prioritize palliative care over aggressive treatment. This case illustrates the complexity of managing cardiac metastases in patients with significant comorbidities. Unlike the first case, where surgical resection was feasible, this patient was not a surgical candidate due to her overall poor prognosis and extensive disease burden.

## 4. Conclusion

These two cases highlight the diagnostic and therapeutic challenges of cardiac metastasis in advanced malignancies. The clinical course is often dictated by the extent of systemic disease and the patient's overall health status, requiring a personalized multidisciplinary treatment approach. Despite advancements in systemic therapies like immunotherapy, cardiac metastases continue to present significant challenges, particularly when complicated by comorbid conditions or immune-related toxicities. Further research is needed to establish more definitive guidelines for managing metastatic cardiac tumors and optimizing outcomes for affected patients.

## Figures and Tables

**Table 1:** Summary of common tumors that metastasize to the heart, including their presentation, mode of metastasis, diagnosis, treatment/prognosis, risk factors, and cardiac complications.

**Tumor type**	**Percentage metastasizing to the heart**	**Presentation**	**Mode of metastasis**	**Diagnosis**	**Treatment/prognosis**	**Risk factors**	**Cardiac complications**	**Location of metastasis**
Lung cancer	30%–40%	Persistent cough, chest pain, shortness of breath, hemoptysis, hoarseness, weight loss, and fatigue	Lymphatic, hematogenous, and direct extension	Imaging (chest x-ray, CT scan, and PET scan) and biopsy	Surgery, chemotherapy, radiation therapy, targeted therapy, and immunotherapy; generally poor prognosis	Smoking, secondhand smoke, radon, asbestos, air pollution, and family history	Pericardial effusion, cardiac tamponade, arrhythmias, heart failure, conduction abnormalities, myocardial infarction, sudden cardiac death, and stroke	Pericardium, epicardium, myocardium, and endocardium
Breast cancer	10%–12%	Lump in breast or underarm, skin changes, nipple changes, and nipple discharge	Lymphatic	Mammography, ultrasound, and biopsy	Surgery, chemotherapy, radiation therapy, hormonal therapy, and targeted therapy; prognosis varies	Increasing age, family history, genetic mutations, early menstruation, late menopause, nulliparity, obesity, and alcohol consumption	Pericardial effusion, cardiac tamponade, arrhythmias, and heart failure	Pericardium and epicardium
Leukemia	30%–44%	Fatigue, fever, frequent infections, shortness of breath, easy bleeding/bruising, bone/joint pain, swollen lymph nodes, and enlarged spleen/liver	Hematogenous	Complete blood count (CBC), bone marrow biopsy, and imaging (CT scan and MRI)	Chemotherapy, targeted therapy, radiation therapy, and stem cell transplant; prognosis varies	Exposure to radiation, chemicals, viruses, and family history	Pericardial effusion, cardiac tamponade, arrhythmias, and heart failure	Myocardium and endocardium
Lymphoma	13.6%	Swollen lymph nodes, fatigue, fever, night sweats, and weight loss	Lymphatic and hematogenous	Physical exam, biopsy, and imaging (CT scan and PET scan)	Chemotherapy, radiation therapy, immunotherapy, targeted therapy, and stem cell transplant; prognosis varies	Weakened immune system, infections, and family history	Pericardial effusion, cardiac tamponade, arrhythmias, and heart failure	Pericardium and myocardium
Melanoma	65%	Skin lesions and symptoms related to metastatic sites	Hematogenous	Biops and, imaging (CT scan and PET scan)	Surgery, chemotherapy, radiation therapy, targeted therapy, and immunotherapy; prognosis varies	Sun exposure, fair skin, family history, and multiple moles	Myocardial or endocardial metastasis, arrhythmias, and heart failure	Myocardium and endocardium
Esophageal cancer	1%	Difficulty swallowing, chest pain, and weight loss	Direct extension	Endoscopy, biopsy, and imaging (CT scan and PET scan)	Surgery, chemotherapy, radiation therapy, and chemoradiation; generally poor prognosis	Smoking, alcohol consumption, Barrett's esophagus, and obesity	Pericardial effusion, cardiac tamponade, arrhythmias, and heart failure	Pericardium and myocardium
Kidney cancer	1%	Blood in urine, flank pain, abdominal mass, weight loss, and fatigue	Hematogenous and direct extension (tumor thrombus)	Imaging (CT scan and MRI) and biopsy	Surgery, targeted therapy, and immunotherapy; prognosis varies	Smoking, obesity, high blood pressure, family history, and genetic conditions	Cardiac tamponade, arrhythmias, and heart failure	Right atrium (via tumor thrombus), myocardium, and endocardium
Ovarian cancer	10.3%	Abdominal bloating, pelvic pain, urinary urgency/frequency, difficulty eating, and weight loss	Hematogenous and lymphatic	Pelvic exam, imaging (ultrasound and CT scan), and biopsy	Surgery, chemotherapy, and targeted therapy; prognosis varies	Family history, genetic mutations, older age, nulliparity, and endometriosis	Pericardial effusion, cardiac tamponade, arrhythmias, and heart failure	Pericardium and epicardium

## Data Availability

The data generated in this study are available upon request from the corresponding author.
